# Splenic artery transposition for hepatic arterial reconstruction in conversion surgery of an initially unresectable, locally advanced pancreatic cancer after gemcitabine/nab-paclitaxel: A case report

**DOI:** 10.1016/j.ijscr.2020.12.041

**Published:** 2020-12-23

**Authors:** Hideharu Tanaka, Hisashi Imai, Toshiya Higashi, Katsutoshi Murase, Nobuhisa Matsuhashi, Kazuhiro Yoshida

**Affiliations:** a Department of Surgical Oncology, Gifu University Hospital, 1-1 Yanagido, Gifu, Gifu, 501-1194, Japan; b Department of General and Cardiothoracic Surgery, Gifu University Hospital, 1-1 Yanagido, Gifu, Gifu, 501-1194, Japan

**Keywords:** Conversion surgery, Pancreatic cancer, Hepatic arterial resection, Case report

## Abstract

**Introduction and importance:**

Recent advances in chemotherapy and chemoradiotherapy allow performance of conversion surgery by improving tumor shrinkage in select patients with initially unresectable locally advanced pancreatic cancer (LAPC), thereby providing curative potential. The number of conversion surgeries requiring arterial reconstruction for select patients with initially unresectable LAPC following favorable responses is expected to increase, so providing effective options for safe arterial reconstruction is critical.

**Case presentation:**

Herein we report a case of successful conversion surgery for initially unresectable LAPC with splenic artery transposition for hepatic arterial reconstruction after gemcitabine/nab-paclitaxel (GnP). A 71-year-old woman was referred to our hospital for evaluation of a pancreatic head mass after developing diabetes. She was diagnosed with unresectable LAPC, which was in wide contact with the common hepatic artery (CHA), proper hepatic artery (PHA), and splenic artery (SA). She received GnP, and after 6 cycles, durations of disease control and normalization of serum carbohydrate antigen 19-9 (CA19-9) exceeded 7 months. She underwent radical subtotal stomach-preserving pancreaticoduodenectomy with CHA-PHA and portal vein (PV) resection (SA-right hepatic artery anastomosis/PV-superior mesenteric vein direct end-to-end anastomosis). Histopathological examination revealed R0 resection with a histological response of Evans grade IIB. No signs of tumor recurrence have been observed for 14 months postoperatively.

**Clinical discussion:**

No consensus has been reached regarding the optimal treatment regimen, duration, or criteria for conversion surgery in patients with LAPC, especially in cases requiring arterial resection. SA transposition for hepatic arterial reconstruction is generally very consistent, easily accessible, and offers adequate length and diameter for successful arterial anastomosis.

**Conclusion:**

Even for a SA initially in contact with the tumor, SA transposition for hepatic artery reconstruction is a safe and effective option when tumor contact disappears due to chemotherapy.

## 1 Introduction

The prognosis of pancreatic cancer (PC) remains dismal, primarily due to the low resectability rate at initial diagnosis. Reportedly, 30–40% of all PC patients present with unresectable locally advanced PC (LAPC) with involvement of nearby vasculature, such as the hepatic, celiac, and superior mesenteric arteries [1]. Recent advances in chemotherapy and chemoradiotherapy have opened pathways to conversion surgery by improving tumor shrinkage in select patients with initially unresectable LAPC, offering curative potential [2,3]. No consensus has yet been achieved regarding the outcomes and clinical implications of pancreatectomy with combined arterial resection, especially with respect to conversion surgery. However, the number of conversion surgeries requiring arterial reconstruction for select patients with initially unresectable LAPC following favorable responses is expected to increase, so provision of effective options for safe arterial reconstruction is critical. Herein we describe a case of successful conversion surgery for initially unresectable LAPC with splenic artery transposition for hepatic arterial reconstruction following treatment with gemcitabine/nab-paclitaxel (GnP). This work has been reported in accordance with the SCARE 2020 criteria [4].

## 2 Case report

**Fig. 1:**
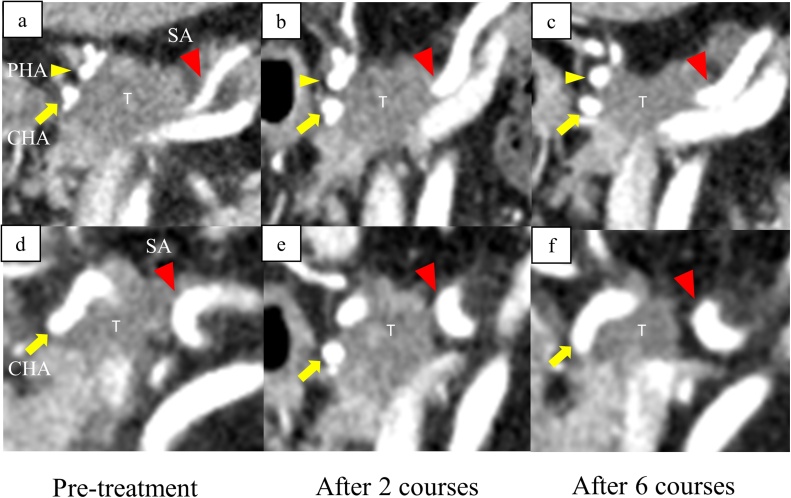
Images from pre- and post-chemotherapy coronal enhanced computed tomography. T: tumor; yellow arrow: CHA; yellow arrowhead: PHA; red arrowhead: SA. a, d) Before GnP chemotherapy. The tumor is in wide contact with the CHA, PHA, and SA. b, e) After 2 cycles of GnP. Distance between the tumor and SA is slightly increased. c, f) After 6 cycles of GnP. The tumor has shrunk, and tumor contact with the SA has disappeared, but contacts with the CHA and PHA remain.

A 71-year-old woman was referred to our hospital for evaluation of a pancreatic head lesion that had been identified incidentally on abdominal ultrasonography after she had developed diabetes. She had hypertension and underwent an appendectomy when she was 30 years of age. She was taking an oral diabetic agent and an anti-hypertensive drug. There was no family history of pancreatic cancer or genetic disorders. The results of the physical examination were unremarkable. Laboratory analyses revealed high levels of hemoglobin A1c (7.5%) and tumor markers, including carbohydrate antigen 19-9 (CA19-9) (228.2 U/mL) and Span-1 (43.0 U/mL). Enhanced multidetector-row computed tomography (CT) revealed a 29-mm hypovascular tumor in the pancreatic head, in wide contact with the common hepatic artery (CHA), proper hepatic artery (PHA), and splenic artery (SA) (Fig. 1a,d). Endoscopic ultrasonography-guided fine needle aspiration cytology was performed, and pathological findings revealed pancreatic adenocarcinoma. PC was diagnosed from these findings based on the 8th edition of the UICC criteria as T4N0M0, Stage III, and was categorized as unresectable LAPC.

The patient underwent chemotherapy with GnP (gemcitabine 1000 mg/m^2^; nab-paclitaxel 260 mg/m^2^) on days 1, 8, and 15 of 28-day cycles. After 2 cycles of induction GnP, serum CA19-9 levels quickly normalized (27.6 U/mL) and remained within the normal range. After 6 cycles, the primary tumor had decreased to 10 mm in diameter, and tumor contact with the SA finally disappeared, but tumor contacts with the CHA and PHA were still observed (Fig. 1). Positron emission tomography (PET) did not reveal any hot spots in the primary tumor or distant metastases. We obtained informed consent from the patient before surgery, and elected to perform conversion surgery with combined CHA-PHA resection to achieve R0 resection.

**Fig. 2:**
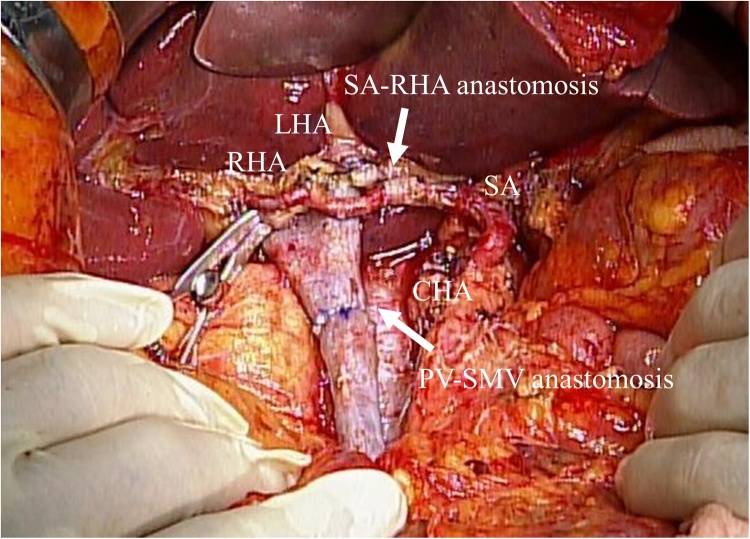
Intraoperative image after tumor resection. Reconstruction involves splenic artery transposition for hepatic arterial reconstruction, and PV-SMV direct end-to-end anastomosis.

Intraoperative findings showed the SA could be separated from the tumor sufficiently, and no change in spleen color was evident even after clamping. However, the PHA and CHA could not be detached from the tumor due to invasion. Based on these findings, we decided to perform CHA-PHA resection with SA transposition for hepatic arterial reconstruction. The left hepatic artery (LHA) was smaller than the right hepatic artery (RHA), and intraoperative ultrasonography confirmed RHA-only blood flow was sufficient to cover the bilateral lobes of the liver. Finally, we performed radical subtotal stomach-preserving pancreaticoduodenectomy (SSPPD) with combined CHA-PHA and superior mesenteric vein (SMV)/splenic vein (SV) resection (SA-RHA anastomosis/portal vein (PV)-SMV direct end-to-end anastomosis) (Fig. 2), and Group 1 and 2 lymph node dissections (D2). An expert hepatobiliary-pancreatic surgeon certified by the Japanese Society of Hepatobiliary Pancreatic Surgery performed the SSPPD, and cardiovascular surgeons skilled in vascular surgery performed the SA-RHA and PV-SMV anastomoses.

**Fig. 3:**
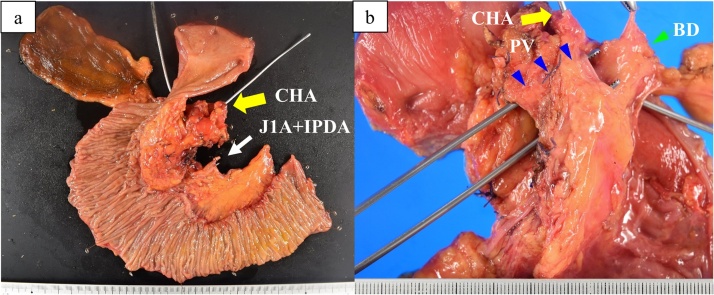
Macroscopic examination of the resected specimen. a) Macroscopic examination of the resected specimen shows a hard mass in the pancreatic head.b) The length of PV resection is 20 mm (blue arrowhead). Yellow arrow: CHA; green arrow: BD (bile duct).

**Fig. 4:**
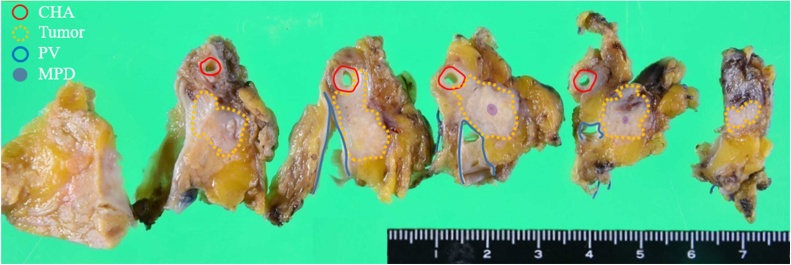
Tumor mapping on the divided surface. A solid tumor (yellow dotted line) in the pancreatic head shows unclear boundaries with the CHA (red line) and PV (blue line) on the divided surface.

**Fig. 5:**
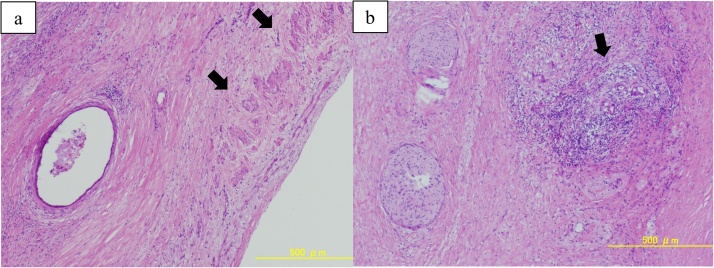
Histopathological findings from hematoxylin and eosin staining. a, b) Histopathological examination reveals degenerated cancer cells extending to the resected CHA adventitia (arrow).

The excised specimen showed a solid tumor in the pancreatic head, and unclear boundaries with the CHA and PV (Fig. 3, Fig. 4). Histopathological examination revealed that the tumor extended to the resected CHA adventitia and resected PV/SV media (Fig. 5). The pathological diagnosis was PC, yp4N1(3/27), M0, ypStage III, based on the 8th edition of the UICC criteria, and grade IIb based on the Evans classification [5]. Histologically, no residual cancer cells were observed in the resected margin, so R0 resection was considered to have been achieved during this conversion surgery.

After surgical treatment, the patient developed liver infarction which was categorized as Grade II in accordance with the Clavien-Dindo classification, in the lateral segment on postoperative day 33. Contrast-enhanced CT showed maintained blood flow in the reconstructed hepatic artery and PV, with no stenosis or occlusion, and improvement was achieved with conservative treatment. The patient was discharged on postoperative day 42, after having received adjuvant chemotherapy with S-1 for 6 months. There were no severe adverse events during adjuvant chemotherapy. The patient had follow-up blood testing and CT scans, and no signs of tumor recurrence have been observed as of 14 months postoperatively.

## 3 Discussion

R0 resection is the only the curative treatment for PC, and can achieve prolonged survival. In recent years, surgery alone has been considered insufficient, and multidisciplinary treatments combining surgical resection and chemotherapy or chemoradiotherapy have been applied to improve prognosis [6,7]. Advances in chemotherapy, such as the FOLFIRINOX and GnP regimens, which have strong antitumor efficacy, and chemoradiotherapy can enable conversion surgery for select patients with initially unresectable LAPC following favorable responses [8]. However, many questions about conversion surgery have remained unanswered.

With respect to optimal preoperative treatment regimens, recent studies comparing FOLFIRINOX and GnP reported that both regimens represent viable options [9,10], but definitive standard treatment protocols, including chemoradiotherapy, remain lacking. The optimal duration of preoperative treatment also remains unclear. A recent review reported that new regimens such as FOLFIRINOX and GnP resulted in tumor shrinkage in a relatively short time (median time to response, 40–50 days), suggesting that a long duration of preoperative treatment is not always necessary for conversion surgery [3]. A number of reports have recently suggested that normalization or marked decreases in serum CA19-9 levels following preoperative treatment represent useful predictors of long-term survival [3,8,11]. In the present case, conversion surgery after GnP with normalized preoperative CA19-9 levels directly impacted survival.

For R0 resection, concomitant vascular resection and reconstruction are often necessary during conversion surgery for LAPC, due to widespread tumor invasion. PV and/or SMV resection has generally been considered to be justified in recent studies from several high-volume centers [12]. In contrast, combined arterial resection for PC remains controversial, due to reported associated high morbidity and mortality rates [13]. Performing pancreaticoduodenectomy (PD) with hepatic artery resection generally requires arterial reconstruction [14], with the most important point being to maintain the required amount of hepatic arterial blood flow while ensuring curability. To achieve this goal, methods such as PHA-left gastric artery end-to-end anastomosis, CHA-PHA end-to-end anastomosis, and CHA-PHA anastomosis using a large saphenous venous graft have been reported [15]. SA transposition for hepatic arterial reconstruction is generally easy to access, and offers an adequate length and diameter for successful arterial anastomosis [16]. In the present case, the merits of SA transposition included: 1) easy access; 2) an autogenous artery; 3) a small-caliber difference between target and graft arteries; 4) close proximity, thereby obviating the need for an interposition graft; and 5) only a single arterial anastomosis. These merits enabled safe hepatic artery reconstruction, and no signs of tumor recurrence had been observed as of 14 months postoperatively. Although the patient developed temporary liver infarction, reconstruction of the resected hepatic artery has been reported as unnecessary, because arterial blood flow is compensated from the preserved hepatic artery and the function of various collaterals [17]. In addition, regarding the possibility of impaired blood flow in the residual pancreas and spleen due to SA dissection, no clinical problems were encountered, because blood flow was donated from the left gastric artery, the stomach wall, and the middle colic artery [18,19]. As of the time of writing, the patient has experienced no relapses of liver infarction and no problems with blood flow in the residual pancreas or spleen.

Although few reports have described patients who underwent PD with combined arterial resection for PC, Bachellier et al. analyzed patients who underwent arterial resection with concomitant pancreatectomy including PD, and reported an acceptable overall mortality rate of 5.1% and median overall survival of 13.7 months [14]. Although these survival data were unsatisfactory, 10 patients survived >3 years. This implies that select patients may benefit from pancreatectomy, including PD with combined arterial resection, and can achieve prolonged survival. However, no well-established indications have been identified for the selection of patients who are most likely to benefit. Considering the high morbidity and mortality rates for this surgical procedure, especially in conversion surgery, combined arterial resection should be performed only in highly experienced centers under the careful scrutiny of multidisciplinary treatment teams [3].

## 4 Conclusions

Further studies are necessary to evaluate and determine the optimal treatment regimen, duration, and criteria for conversion surgery and resection, especially for cases requiring arterial resection. Even for a SA initially in contact with the tumor, SA transposition for hepatic artery reconstruction is a safe and effective option when tumor contact disappears due to chemotherapy.

## Declaration of Competing Interest

The authors declare that they have no competing interests.

## Funding

This report did not receive any specific grant from funding agencies in the public, commercial, or not-for-profit sectors.

## Ethical approval

This study is exempt from ethical approval in our institution.

## Consent

Written informed consent was obtained from the patient for publication of this case report and accompanying images. A copy of the written consent is available for review by the Editor-in-Chief of this Journal on request.

## Author contribution

HI (Expert hepatobiliary-pancreatic surgeon certified by the Japanese Society of Hepatobiliary Pancreatic Surgery) and HT performed the surgery on this patient. IH, HT, KM, and TH managed the postoperative course of the patient. HT and HI wrote the manuscript. All authors have read and approved the final manuscript.

## Registration of research studies

Our case report is not first-in-man. Therefore, in accordance with the Guidance of Research Registry, we did not register our case report at http://www.researchregistry.com.

## Guarantor

Hideharu Tanaka (Corresponding author and Guarantor).

## Provenance and peer review

Not commissioned, externally peer-reviewed.

## Availability of data and materials

Not applicable.
